# Hub overload and failure as a final common pathway in neurological brain network disorders

**DOI:** 10.1162/netn_a_00339

**Published:** 2024-04-01

**Authors:** Cornelis Jan Stam

**Affiliations:** Clinical Neurophysiology and MEG Center, Department of Neurology, Amsterdam Neuroscience, Amsterdam UMC, Vrije Universiteit Amsterdam, Amsterdam, The Netherlands

**Keywords:** Hubs, Stroke, Epilepsy, Multiple sclerosis, Alzheimer’s disease, Cascading failure

## Abstract

Understanding the concept of network hubs and their role in brain disease is now rapidly becoming important for clinical neurology. Hub nodes in brain networks are areas highly connected to the rest of the brain, which handle a large part of all the network traffic. They also show high levels of neural activity and metabolism, which makes them vulnerable to many different types of pathology. The present review examines recent evidence for the prevalence and nature of hub involvement in a variety of neurological disorders, emphasizing common themes across different types of pathology. In focal epilepsy, pathological hubs may play a role in spreading of seizure activity, and removal of such hub nodes is associated with improved outcome. In stroke, damage to hubs is associated with impaired cognitive recovery. Breakdown of optimal brain network organization in multiple sclerosis is accompanied by cognitive dysfunction. In Alzheimer’s disease, hyperactive hub nodes are directly associated with amyloid-beta and tau pathology. Early and reliable detection of hub pathology and disturbed connectivity in Alzheimer’s disease with imaging and neurophysiological techniques opens up opportunities to detect patients with a network hyperexcitability profile, who could benefit from treatment with anti-epileptic drugs.

## INTRODUCTION

An important assumption in clinical neurology has always been that circumscribed brain regions have specific functions and will produce characteristic symptoms and signs if they are affected by brain disease. It is now becoming clear that all these specialized brain regions are interconnected in a complex, well-organized brain network. The fact that the brain is a network matters when it comes to neurological disease, as this may help to understand how supposedly focal lesions can give rise to more diffuse neurological dysfunction, far beyond the site of the initial lesions (Bartolomei et al., 2006; Bassett & Bullmore, 2009; Stam, 2014; van Dellen et al., 2013).

A proper understanding of the implications of the network nature of the brain for neurological disease has only become feasible relatively recently due to the emergence of the new interdisciplinary field of network science (Albert & Barabási, 2002; Strogatz, 2001). Within this field, networks are represented as sets of nodes (also referred to as “vertices”) and links (“edges”) connecting these nodes. This abstract representation of a network is called a graph. Various neuroimaging techniques such as magnetic resonance imaging (MRI), electro-encephalography (EEG), and magneto-encephalography (MEG) can be used to represent empirically observed structural and functional brain networks as graphs. Network science studies the different ways in which the links in a network or graph can be organized. This organization, called the topology of the network, has far reaching consequences for the function of the network, and the nature of the processes that can take place upon the network. Two early major breakthroughs in network science have been the discovery of “small-world” and “scale-free” networks (Barabási & Albert, 1999; Watts & Strogatz, 1998). Small-world networks combine high levels of local connectivity with strong overall integration. This optimal balance between segregation and integration is of paramount importance for the functioning of brain networks (Bullmore & Sporns, 2012). Scale-free networks are even better integrated due to the presence of highly connected hub nodes. Features of small-world and scale-free networks have been discovered in empirical brain networks (Bassett & Bullmore, 2009). The organization of brain networks is relevant for understanding general mechanisms of brain pathology (Fornito et al., 2015). This can be illustrated with the example of hub nodes in brain networks.

A hub in a network is defined as a node with a high level of centrality (Oldham & Fornito, 2019; Zuo et al., 2012). The concept of centrality refers to various graph theoretical measures that quantify the relative importance of a node within a network. The simplest example of centrality is the degree of a node. A node with many links has a high degree centrality. The presence of one or more hubs with a high degree centrality will often reduce the path length between any two nodes considerably. In other words, the presence of hubs is important for the level of integration in a network. If the network is a transport system, a large part of the traffic on the network will be handled by the hubs. For instance, in the case of air traffic, major airports are typical examples of hubs that will handle a very large number of incoming and outgoing flights. In addition to degree centrality, there are many other types of centralities that can also quantify how important a node is within a network. For instance, the betweenness centrality of a node indicates which fraction of all the shortest paths on the network pass through this node. The eigenvector centrality of a node takes into account not only the connectivity of a node itself, but also the connectivity of its neighbors, and that of the neighbors of its neighbors, and so on. Other examples of centrality measures are closeness centrality and Page Rank. Hubs can be defined in terms of one of these centrality measures, or in terms of some combination of two or more different centrality measures. When a network is divided into subnetworks or modules, we can also distinguish connector hubs, which connect their own module to other modules, and provincial hubs, which have mainly connections in their own modules. When a set of hubs are more strongly connected to each other than expected on the basis of their degree this is called a rich club (van den Heuvel & Sporns, 2011).

There is strong evidence for the presence of hubs in brain networks (van den Heuvel & Sporns, 2013). Of interest, it is even possible to identify hub neurons in very simple neural networks such as the nervous system of *C. elegans* (Uzel et al., 2022). However, for the purpose of the present review, the main interest is in large-scale networks of interconnected cortical and subcortical brain regions. At this level the following regions are most often identified as hubs in structural brain networks: precuneus, anterior and posterior cingulate cortex, superior frontal cortex, temporal cortex, lateral parietal cortex, and the insula (van den Heuvel & Sporns, 2013). These hubs have many, often long distance, connections to other brain regions, and are responsible for a very high level of integration or short average path length in healthy brain networks. Analogous to transport networks, hubs in brain networks also handle a disproportionally large part of the information flow taking place in the brain. Related to this, hubs in brain networks are characterized by a high level of glucose consumption and metabolism, high firing rates of neurons, and high expression levels of genes related to ATP production and plasticity (Tomasi et al., 2013). An important downside of this high level of metabolism and neural activity of hubs is their vulnerability to damage. Thus, hubs are crucial for integration and communication in networks, but also constitute weak spots for network damage, which makes them relevant for clinical neurology.

There is evidence that hubs in brain networks are preferentially involved in the pathology of a large variety of neurological as well as psychiatric disorders. In a seminal study Crossley et al. (2014) first identified hub nodes based upon the diffusion tractography imaging (DTI) MRI structural networks of 56 healthy subjects. Next, in a simulation study, they showed that a “targeted attack” on these hub nodes had a much larger disruptive effect on the whole network than a “random attack.” Finally, they constructed gray matter lesion maps, based on meta-analyses of published MRI data on more than 20,000 subjects and 26 different brain disorders. For nine conditions, including Alzheimer’s disease and schizophrenia, they could demonstrate a significant association between the localization of the gray matter lesions and the hubs. This study therefore provides support for the hypothesis that hubs constitute weak spots in brain networks and may be damaged in a wide variety of conditions.

The present review provides an update and extension of the concept of hub vulnerability in neurological disease. I examine recent evidence obtained with imaging techniques such as MRI, EEG, MEG, and positron emission tomography (PET) for involvement of hubs in epilepsy, stroke, multiple sclerosis, and Alzheimer’s disease. These examples are chosen to show how hub involvement has become a common theme across neurological disorders with a very different pathophysiology and spatial distribution (Aben et al., 2019; Chard et al., 2021; Li et al., 2022; Royer et al., 2022). In particular, I explore whether knowledge of hub involvement in these conditions has made any contribution to understanding of underlying pathophysiology, diagnosis, assessment of prognosis, and treatment.

## EPILEPSY

### Introduction

Epilepsy is increasingly considered to be a disorder of brain networks (de Palma et al., 2020). The concept of an epileptic focus as a well circumscribed area of hyperexcitability where seizures originate has been replaced with the more complex notion of an epileptic network, which reflects the dynamic and spatially distributed interaction between multiple brain regions involved in the onset and propagation of seizures (van Diessen et al., 2013a). In addition, there is now also increasing awareness of the role of highly connected hub nodes in epilepsy (Royer et al., 2022). Physiological hubs may be disrupted by seizures arising elsewhere, whereas pathological hubs could potentially contribute to spreading patterns of epileptic activity. Here, I will explore what we currently know about network changes, with a particular focus upon hubs, in epilepsy.

### Generalized Epilepsy

There is no agreement yet on the presence and nature of network changes and hub involvement in generalized epilepsy. There are indications of a loss of structural connectivity in generalized epilepsy (Lee et al., 2020). This loss of structural connectivity is accompanied by a lower global and local efficiency, longer path length and a reorganization of network hubs. With respect to functional brain networks, one EEG study compared untreated subjects with focal or generalized epilepsy with healthy controls (van Diessen et al., 2016). There were no network changes in the group with generalized epilepsy. In the group with focal epilepsy, network changes depended upon the frequency band: in the delta band there was a less integrated network with a low betweenness centrality, whereas a more integrated network was found in the alpha band. Another functional network study based upon resting-state fMRI reported a more regular network topology, increased functional connectivity, and a lack of changes in hubs (Pegg et al., 2021). It could be that MRI is more sensitive to network changes than routine scalp EEG. A pattern of increased functional connectivity and decreased structural connectivity is not necessarily contradictory, and could reflect disturbed structure-function coupling (Chiang et al., 2015; Liu et al., 2022).

### Temporal Lobe Epilepsy: Structural Networks

Focal epilepsy, in general, and temporal lobe epilepsy (TLE), in particular, has been the topic of many network studies. This interest can be explained by the fact the TLE is one of the most frequent types of epilepsy in adults, and often shows insufficient response to anti-epileptic drugs. Consequently, TLE patients are often candidates for epilepsy surgery, and here network aspects of epilepsy may become particularly relevant. Network changes in TLE have been demonstrated with structural MRI, functional MRI, EEG, depth recordings, and MEG. Structural networks can be approximated by computing correlation matrices of the cortical thickness of multiple brain regions as assessed with structural MRI. With this approach a loss of long-distance connectivity and a shift toward more local connectivity has been found (Bernhardt et al., 2011; Yasuda et al., 2015). Both studies also showed an altered hub distribution, which was correlated with an unfavorable outcome after epilepsy surgery. A study with DTI tractography also demonstrated loss of white matter connectivity in temporal, frontal, and parietal areas (Liu et al., 2014a). There were also alterations in the left precuneus, which is one of the major hubs of brain networks. A very large study in 1,021 patients with epilepsy and 1,564 healthy controls showed that atrophy in TLE occurred at the same location as hubs, whereas in generalized epilepsy there was involvement of subcortical hubs (Larivière et al., 2020). Structural networks are thus disrupted in TLE, and these abnormalities extend far beyond the temporal lobe. In addition, hub nodes are involved, within and outside the temporal lobe.

### Temporal Lobe Epilepsy: fMRI

Network changes in TLE have also been studied with resting-state and task fMRI. Resting-state fMRI has shown widespread network changes in TLE (Ridley et al., 2015). In contrast to the findings in structural networks described above, functional networks may show evidence of increased global integration as reflected by higher global efficiency and shorter path length (Mazrooyisebdani et al., 2020). This is in line with the observation that functional network changes do not always correlate with structural network changes in the same subject (Chiang et al., 2015; Douw et al., 2015). In addition to the more widespread changes, a more circumscribed disruption of the language and motor network has been demonstrated in a task fMRI study (Roger et al., 2020). Functional connectivity between the default mode network, or individual hubs within this resting-state network, and other brain regions is disturbed in TLE (Bernhardt et al., 2016; Douw et al., 2015; Frings et al., 2009). One study reported abnormal connectivity between hub nodes within the DMN, accompanied by increased connectivity between the DMN and other brain regions (Douw et al., 2015). The distribution of hubs nodes is also affected in TLE (Lee et al., 2018; Mazrooyisebdani et al., 2020). Disturbed connectivity and altered distribution of hub nodes is associated with seizure frequency and cognitive function (Douw et al., 2015; Mazrooyisebdani et al., 2020). Thus, functional networks are clearly involved in TLE. The abnormalities may extend beyond the temporal lobe and involve hub nodes. The changes are clinically relevant since they correlate with seizure frequency, but they do not correlate very well with structural changes.

### Temporal Lobe Epilepsy: EEG and MEG

In contrast to fMRI, techniques like EEG and MEG can measure neural activity directly with a very high time resolution. This allows a different perspective on network changes in TLE. Resting-state MEG has demonstrated the presence of pathological hubs in left temporal areas and the right posterior cingulate cortex (Jin et al., 2015a). In TLE, changes in resting-state source-space MEG networks can be used to determine the lateralization of the epileptogenic zone (Pourmotabbed et al., 2020). Two neurophysiological studies have examined the spatial relation between the conventionally defined epileptic focus and nearby pathological hubs. In a study with depth recordings in the temporal lobe the epileptogenic zone, defined in terms of epileptiform discharges and the presence of high-frequency oscillations (HFOs), showed low levels of node centrality (van Diessen et al., 2013b). In addition, HFO count was negatively correlated with measures of hubness. A similar conclusion was reached in a MEG study with virtual electrodes (Nissen et al., 2016). In this study measures of node centrality increased with larger distance from the epileptic focus, defined in terms of interictal epileptiform discharges and HFOs. Both studies show that pathological hubs do not coincide with the epileptic focus, although they are often found in its neighborhood. These findings have been interpreted in terms of a functional disconnection of the epileptic focus in the interictal state.

### Hubs and Seizure Dynamics

In view of the possible importance of hubs in epilepsy it is helpful to know what happens to hubs of functional brain networks in the preictal, ictal, and postictal period. In focal cortical dysplasia, interictal MEG recordings have shown an increase in the efficiency of cortical hubs and the betweenness centrally of the posterior cingulate cortex (Jin et al., 2015b). Stereo EEG recordings show that during seizures the lesion nodes act as functional hubs involved in the start, spreading, and end of epileptic seizures (Varotto et al., 2012). Seizures may also be associated with multiple, independent hubs characterized by activity in the high gamma band (Tobochnik et al., 2022). Synchronization within and between the epileptic focus and the surrounding epileptic networks can undergo complex changes in the transition from preictal to ictal states (Khambhati et al., 2015). These observations suggest that the epileptic focus is disconnected from nearby pathological hubs in the interictal/preictal state (Nissen et al., 2016; van Diessen et al., 2013b). The transition to an ictal state could then be understood as the establishment of connections between the epileptic focus and surrounding pathological hubs, which subsequently could play a role in the spreading of seizure activity to other parts of the network. Further support for this interpretation comes from a simulation study that shows the importance of connections between the epileptic focus and nearby hubs nodes for seizure spreading (Nissen et al., 2021).

### Network Hubs and Epilepsy Surgery Outcome

If hubs are involved in the spreading of seizures through the brain, one would expect that surgical removal of hub nodes would be associated with a postoperative reduction in seizures. This idea has been studied at two levels. A number of studies have tried to correlate the organization of preictal brain networks with surgery outcome. A few other studies have tried to establish a direct relationship with hub removal and seizure reduction.

There is considerable evidence that preictal brain network organization is related to postoperative seizure reduction (Bernhardt et al., 2011; Douw et al., 2015; Gleichgerrcht et al., 2020; Grobelny et al., 2018; Ofer et al., 2019; van Dellen et al., 2014). All of these studies suggest that features of hubs, such as their level of structural and functional connectivity, or their spatial reorganization, are related to outcome after surgery. However, these studies do not yet allow for simple conclusions with respect to the desirability of hub removal in epilepsy. One somewhat atypical study even suggests that hubs could protect against seizures and that their removal would be a bad thing (Grobelny et al., 2018). A promising approach for clinical application is the use of machine learning applied to structural and network features derived from MRI in TLE and controls (Gleichgerrcht et al., 2020). With this approach the authors were able to predict surgical outcome on the basis of regional atrophy patterns in combination with a typical hub measure (betweenness centrality of the parahippocampal and superior temporal gyri).

A few studies have addressed explicitly the question whether surgical removal of hubs is associated with a favorable outcome after epilepsy surgery. Zubler et al. (2015) investigated transfer entropy, a measure of directed connectivity, in 198 seizures of 27 patients. In this study a higher percentage of hub nodes in the resection area was associated with a good outcome. In a study using MEG source-space functional connectivity, hubs were located in the resection area in 9 out of 14 subjects in the seizure-free group, and in none of 8 subjects who were not seizure free (Nissen et al., 2017). However, in a follow-up study in 94 patients, 64 of whom were seizure free, MEG measures of power, functional connectivity, and betweenness centrality were not effective in predicting the epileptogenic zone and outcome (Nissen et al., 2018). Ramaraju et al. (2020) also used MEG to determine the degree centrality of nodes in a functional brain network. This study was more successful and showed that the average degree centrality in resected as compared to not resected nodes was much higher in the group of patients with a good outcome.

### Conclusion

**Figure 1.**
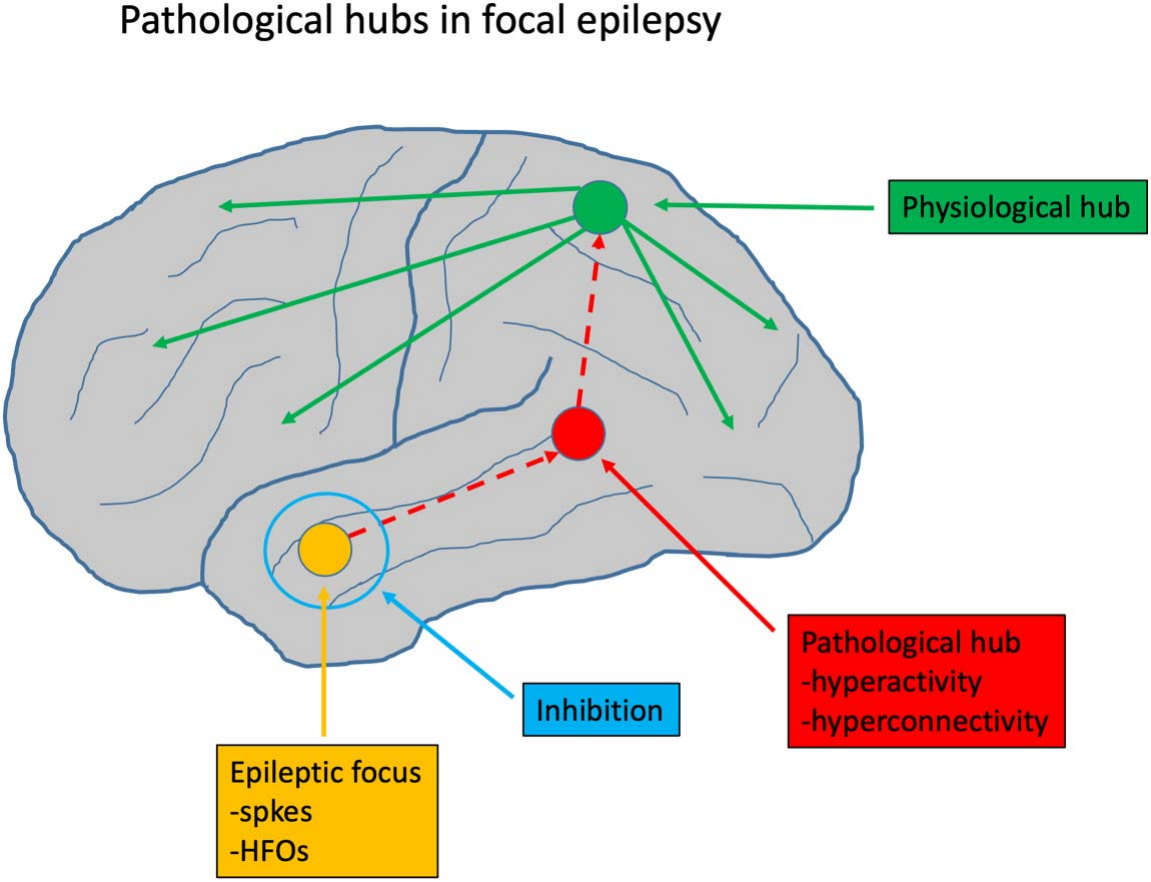
Possible role of pathological hubs in focal epilepsy. The primary epileptic focus, indicated in orange, is a local brain area with an abnormally high excitability. This can show up in EEG or MEG recordings as focal interictal epileptiform discharges (spikes) or high-frequency oscillations (HFOs). Often such an epileptic focus is surrounded by a zone of inhibition, indicated by the blue circle. If the local inhibition breaks down, abnormal activity from the focus can spread to a nearby pathological hubs. This is a region that has developed abnormally high structural and functional connectivity due to damage and partial recovery. When abnormal activation reaches the pathological hub, it may spread to other regions, including the highly connected physiological hubs such as the precuneus and posterior cingulum. From there, abnormal activation can rapidly spread to the rest of the brain, causing a generalized seizure. This schema explains how pathological hubs are different from the primary focus, have a distinct neurophysiological signature (hyperconnectivity instead of epileptiform discharges), and constitute an important relay station in the process of seizure generalization. Removal of pathological hubs may improve outcome in epilepsy surgery.

There is considerable evidence for changes in structural and functional brain networks, especially focal epilepsy syndromes. These network changes extend far beyond the epileptic focus. The network changes almost always involve cortical as well as subcortical hub nodes. Physiological hubs, notably in the DMN, may be disrupted, and new, pathological hubs may emerge. This hub reorganization correlates with cognitive dysfunction and seizure frequency. Pathological hubs probably do not coincide with the epileptic focus, but are often located nearby, and their removal could have a favorable effect on outcome after epilepsy surgery (Figure 1).

## STROKE

### Introduction

Like epilepsy stroke represents another major neurological condition where brain networks are involved, and where highly connected hub nodes play a key role. However, there are a few differences that need to be taken into account. In epilepsy pathological hubs can be directly involved in the pathophysiological process by facilitating the spreading of epileptic activity, whereas damage to physiological hubs may be responsible for cognitive dysfunction, especially in chronic epilepsy. In stroke the major problem is the direct effect of stroke lesions on hubs. Such involvement can have a major impact on network organization and the development of motor and nonmotor symptoms (Aerts et al., 2016). In addition, a proper understanding of network effects in stroke also requires one to take into account effects of plasticity and long-term network reorganization.

### Evidence for Network Reorganization and Hub Involvement in Stroke

There is considerable evidence for network reorganization and hub involvement in stroke. In a study of structural brain networks in chronic stroke patients, the betweenness centrality of network nodes in the orbitofrontal regions was decreased, whereas the betweenness centrality of the parieto occipital hub areas was increased, possibly due to compensatory effects (Shi et al., 2013). Hub disruption after stroke has been demonstrated in the contralateral hemisphere, and can be quantified by the “Hub disruption index” (Termenon et al., 2016). Expansion of the gray matter in brain regions such as the precuneus, overlapping with cortical hub regions in healthy individuals, has been demonstrated in the acute phase poststroke (Chen et al., 2021). This was accompanied by gray matter shrinkage in other, nonhub regions. An important determinant of the impact of stroke lesions on brain networks is the number of fibers that pass through the lesion (Egger et al., 2021). This may explain the disproportional effects of lesions involving hubs, and is somewhat reminiscent of the concept of “strategic infarctions.” Damage to the hubs of the default mode network in stroke patients with impaired consciousness has recently been demonstrated by EEG as well (Serban et al., 2022). While many studies focus on hemispheric stroke, there is also evidence that subcortical stroke induces widespread network changes (Yan et al., 2022).

### The Impact of Hub Lesions on Outcome Prediction in Stroke

One can expect that the impact of a stroke lesion on motor and nonmotor function will depend not only on its size, but also to an important extent to its overlap with one or more important hub regions in the brain network. There is now increasing evidence that hub involvement in stroke lesions has important consequences for prognosis of motor and nonmotor functions.

Several studies showed that involvement of the hub nodes in the stroke lesions correlates with motor impairment, impaired behavioral recovery, and spatial neglect (Chen et al., 2021; Sotelo et al., 2020; Wang et al., 2021a). The importance of network properties for outcome prediction was confirmed in the study of Ktena et al. (2019), which showed that inclusion of graph properties such as path length and rich club features improved prediction of functional outcome. Ischemic stroke in the pons is also associated with an unfavorable prognosis for motor recovery (Olafson et al., 2021). Even when there is not yet a real stroke but only a transient ischemic attack, functional network changes that involve the default mode network and the posterior cingulate cortex already predict a higher probability of recurrence of transient ischemic attacks (Zhu et al., 2019).

Since hub involvement in stroke frequently entails widespread network changes, one can expect cognition to be even more at risk than basic motor and sensory function. Poststroke dementia is more likely when the lesion involves major hub nodes of fMRI functional brain networks or disrupts cholinergic pathways (Lim et al., 2014). Poststroke aphasia is more likely if structural hub nodes in the left hemisphere are damaged. Poststroke depression has been associated with pathological hyperconnectivity of hubs in the default more network (Liang et al., 2020). Aben et al. (2019) introduced the “lesion impact score,” which combines information on hub status of healthy brain networks with node involvement in a stroke lesion. With this lesion impact score, they were able to predict recovery of poststroke cognitive dysfunction (2019).

### Conclusion

Where a stroke occurs in the brain, and how large it is, does not depend directly upon the properties of the underlying brain networks. However, and this is an important message, the extent to which a stroke lesion overlaps with one or more critical hubs of brain networks has major consequences for the severity and long-term recovery of motor and especially nonmotor symptoms after stroke. A promising development is the introduction of relatively simple measures such as the hub disruption index and the lesion impact score, which may enable reliable prediction of prognosis after stroke in a clinical setting (Aben et al., 2019; Termenon et al., 2016). While the static effects of stroke on brain networks are fairly well understood, a major challenge for future studies is to gain a better understanding of the effect of different types of plasticity on poststroke network reorganization.

## MULTIPLE SCLEROSIS

### Introduction

In multiple sclerosis there is often a striking discrepancy between clinical symptoms and signs on the one hand, and abnormalities on structural imaging, in particular, white matter lesions on MRI, on the other hand. This problem, referred to as the clinical-radiological paradox, is particularly clear in the case of cognitive dysfunction and fatigue, which are difficult to relate to isolated, local lesions. Network studies in MS have been motivated in part by the hope that they could contribute to a solution of this problem (Chard et al., 2021). It is of particular interest that some reviews have pointed to the importance of hubs in network failure in MS (Schoonheim et al., 2022; Stampanoni Bassi et al., 2019). Here I investigate the evidence for hub involvement in structural and functional networks in MS, and the implications of this for cognitive dysfunction and fatigue.

### Structural Networks in MS

Many studies have demonstrated disruption of structural networks in MS. The strength of structural connections is almost invariably decreased (Cho et al., 2018; Llufriu et al., 2016; Pagani et al., 2020; Shu et al., 2018a; Stellmann et al., 2020). Long-distance fibers are affected, in particular (Meijer et al., 2020). Cortical hub nodes such as the precuneus are also a major target of structural network changes, even in the early phase (Cho et al., 2018; Shu et al., 2018a; Stellmann et al., 2020). Sometimes, a combination of loss of preexisting physiological hubs and emergence of new pathological hubs can be found, which points to a reorganization of the structural network (Llufriu et al., 2016; Pagani et al., 2020). In addition to cortical hubs, subcortical hub structures such as the thalamus and basal ganglia are also involved (Eshaghi et al., 2018; Tewarie et al., 2015). Atrophy of deep gray matter volume is a major determinant of progression of clinical disability (Eshaghi et al., 2018). However, tracts of white matter that pass through these subcortical hub structures are relatively protected against MS lesions (Clarke et al., 2022a). Also, and perhaps counterintuitively, atrophy of the thalamus can coincide with increased functional connectivity between the cortex and the thalamus (Tewarie et al., 2015).

### Functional Connectivity in MS

Functional connectivity in MS has been mostly studied with resting-state fMRI. Compared to the findings in structural network studies, the results of functional connectivity are more variable. Some studies report a loss of functional connectivity and hubs (Koubiyr et al., 2020; Rocca et al., 2016). The study of Koubiyr et al. (2020), which characterized network reorganization with the hub disruption index, showed a progression of the hub related loss of functional connectivity after a follow-up period of 1 year. Reorganization of functional network structure with loss of preexisting and emergence of new hubs was also evident in the study of Rocca et al. (2016). However, other studies in a large sample of 332 MS patients and 96 healthy controls reported an increase in voxel-based degree and eigenvector centrality, especially in the DMN, and an increase of functional connectivity between the DMN and the fronto-parietal network and the rest of the brain in a subset of cognitively impaired patients (Eijlers et al., 2017; Meijer et al., 2017). A lower structural and higher functional connectivity of hubs at baseline was reported in a trial that investigated the effect of aerobic training on brain networks in MS (Stellmann et al., 2020). Of interest, after 3 months of exercise there was a widespread increase in both structural as well as functional connectivity in the treatment group. A complex pattern of increased and decreased functional connectivity has also been reported (Rocca et al., 2018). These connectivity changes were correlated with various clinical manifestations.

Changes in functional connectivity have also been studied with MEG. Early studies showed an increase in functional connectivity, in particular, in the theta band and above the parietal regions (Hardmeier et al., 2012; Schoonheim et al., 2013). In both studies, decreased functional connectivity was also reported in frequencies outside the theta band. A limitation of these early studies is the fact that the analysis was done in signal space, and analysis of connectivity was not corrected for the influence of signal spread. More recent studies determined functional connectivity in source space and corrected for signal spread (Sjøgård et al., 2021; Tewarie et al., 2015). Tewarie et al. (2015) reported a loss of cortical integration of functional MEG networks. This was correlated with thalamic atrophy and increased fMRI thalamocortical connectivity. Remarkably, in the same study cortical fMRI connectivity in MS was not significantly different from healthy controls. A reduction of functional connectivity of the DMN was reported in the MEG study of Sjøgård et al. (2021). In this study changes in specific connections and subnetworks could be related to disability and disease duration.

### Network Changes, Cognition, and Fatigue in MS

Network changes and hub involvement in MS are of special interest in relation to cognitive dysfunction and fatigue. Diminished strength of structural connections, loss of hubs, and network reorganization are all correlated with cognitive dysfunction, in particular, with respect to attention and executive function (Cho et al., 2018; Llufriu et al., 2016; Shu et al., 2018a). Structural networks can also contribute to fatigue in MS (Fleischer et al., 2022). In fMRI studies, cognitive dysfunction has been related to both increases as well as decreases of functional connectivity (Eijlers et al., 2017; Meijer et al., 2017, 2020; Rocca et al., 2016). Functional connectivity changes in hub involvement have also been implicated in a loss of cognitive reserve and increased fatigue in MS (Bizzo et al., 2021; Jaeger et al., 2019). In the study of Hardmeier et al. (2012), changes in theta band parietal centrality were correlated with cognitive dysfunction. Loss of integration of cortical networks and disturbed connectivity of the DMN is also associated with cognitive disturbances (Sjøgård et al., 2021; Tewarie et al., 2015). Many of the studies above are cross-sectional. This raised the important question, to what extent network changes can predict future cognitive dysfunction? The study of Nauta et al. (2021) showed that changes in MEG network organization in the delta and theta band could predict cognitive dysfunction after a follow up of 5 years, even after correction for the effect of structural changes.

### Conclusion

Recent studies all confirmed the involvement of hubs and disturbances of connectivity in MS. In addition to damage to the classic hubs in the posterior default mode network, also other hubs in subcortical and thalamic regions are implicated. While structural connections are always weakened, functional connectivity may be increased or decreased. Possibly, increased functional connectivity may represent and early phase of compensation or disinhibition. Importantly, structural and functional connectivity may change independently, and perhaps in different phases of the disease. I will address a possible scenario to explain these findings in the section about the hub overload and failure framework. An important conclusion is that damage to hubs, and the accompanying network reorganization, have important implications for present and future cognitive dysfunction and fatigue. This suggests that robust measures of hub failure in MS could become potentially valuable as clinical biomarkers.

## ALZHEIMER’S DISEASE

### Introduction

Alzheimer’s disease has been a major topic for network studies from the start (Stam et al., 2007; Tijms et al., 2013). The selective vulnerability of hub areas was discovered in an early stage. Of particular interest is the fact that damage to hub nodes has been related to the deposition of pathological proteins such as amyloid-beta and tau (Buckner et al., 2009; Li et al., 2022). In addition, recent studies suggest a relation between the pathophysiological process of network hyperexcitability and early damage to hubs. Here I review evidence from structural, functional, and molecular imaging studies for specific involvement of hubs in AD and discuss how this may be related to amyloid-beta and tau.

### Structural Brain Networks

MRI studies that reconstruct structural networks from correlations between cortical thickness of different brain areas have shown a loss of long-distance interregional connections and damage to cortical hubs (Canal-Garcia et al., 2022; Voevodskaya et al., 2018). Damage to hub nodes can precede atrophy of the involved brain regions (Voevodskaya et al., 2018). A loss of structural connectivity and nodal efficiency in hub nodes has also been demonstrated with MRI tractography (Lo et al., 2010; Shu et al., 2018b). In these studies, global efficiency, which depends strongly on long-distance connections, was also decreased, and network changes correlated with behavioral performance and memory disturbances. In mild cognitive impairment (MCI) and asymptomatic subjects, loss of structural connectivity has been detected in areas outside the usual hub nodes (Clarke et al., 2022b; Kim et al., 2019).

### Functional Brain Networks

Selective loss of functional connectivity of hub nodes has been reported in many resting-state fMRI studies (Badhwar et al., 2017; Brier et al., 2014; Drzezga et al., 2011; Ibrahim et al., 2021; Minati et al., 2014; Wang et al., 2021b). This loss of functional connectivity has even been found in amyloid positive asymptomatic subjects (Brier et al., 2014; Drzezga et al., 2011). Loss of functional connectivity progresses during the course of AD (Kundu et al., 2019). Several studies point out that the loss of functional connectivity in AD not only specifically involves hubs, but also long-distance connections (Dai et al., 2015; Delli Pizzi et al., 2019; Liu et al., 2014b). However, an increase in resting-state fMRI functional connectivity has also been reported (Delli Pizzi et al., 2019; Li et al., 2021; Sui et al., 2015). In the study of Delli Pizzi et al. (2019), MCI patients with increased functional connectivity had a higher probability of developing dementia than patients with low functional connectivity. Changes in functional connectivity have also been related to the e4 allele of the APOE gene, which is one of the most important risk factors of sporadic Alzheimer’s disease. Wang et al. (2015) showed that AD patients who were positive for the e4 allele had a disturbed functional network organization with a loss of connectivity from the posterior default mode network. However, in an fMRI study during a visual scene discrimination task, young asymptomatic subjects who were e4 positive showed increased structural connectivity and activation of the posterior default mode network (Li et al., 2021). One might hope that changes in hub functional connectivity, in contrast to structural damage, could be potentially reversible. One small trial in 16 subjects with MCI and 16 healthy controls showed an increase in functional connectivity in the posterior cingulum and precuneus in the MCI group after 12 weeks of walking exercise (Chirles et al., 2017). Unfortunately, a more recent trial in 45 AD patients did not show an effect of exercise on hub functional connectivity (Musaeus et al., 2022).

### PET Studies

PET has been used extensively in AD, either as a separate modality, or in combination with structural or functional MRI. An early fluordesoxyglucose (FDG) PET study showed a decrease of betweenness centrality in several hubs in the default more network in MCI and AD (Seo et al., 2013). FDG PET showed a lower level of glucose consumption in hub nodes, which correlated with a loss of fMRI functional connectivity (Mutlu et al., 2016; Zhang et al., 2021).

Of particular interest for understanding the pathophysiology of AD are studies that relate amyloid PET with fMRI. These studies confirm that AD pathology primarily affects hub nodes (Li et al., 2022). In a classic study, Buckner et al. (2009) showed a correlation between levels of amyloid deposition and fMRI-based functional hub regions. This relation between amyloid deposition and resting-state fMRI functional connectivity in hub regions was confirmed in more recent studies (Mutlu et al., 2017; Myers et al., 2014; Sintini et al., 2021). However, it should be stressed that the relation between amyloid-beta and functional connectivity may be complicated by the possibility that amyloid deposition starts in highly connected hubs, but may subsequently give rise to structural damage that will reduce this high connectivity (Myers et al., 2014). I will come back to this in the discussion of the hub overload and failure scenario.

While tau may be less specific for AD than amyloid, it correlates more closely with clinical symptoms and cognitive dysfunction. There is evidence for increased tau binding in the precuneus and posterior cingulum in AD (Yokoi et al., 2018). This correlates with a loss of functional connectivity from the posterior cingulum. In another study, tau pathology was associated with hub nodes with a high level of functional connectivity (Cope et al., 2018). These authors also stressed that in a later stage tau may induce neuronal damage and a decrease in the level of connectivity. Such a late stage could explain why high tau levels were associated with low functional connectivity in the study of Sintini et al. (2021). In cognitively intact Presenilin 1 carriers, tau was associated with decreased segregation and integration of the default mode network (Guzmán-Vélez et al., 2022).

### EEG

Numerous EEG and MEG studies addressed network changes in MCI and AD. Here the focus is on studies that specifically looked at changes in hubs. In an EEG study in 318 AD patients, disease severity correlated with a loss of functional connectivity in the alpha band (Engels et al., 2015). Betweenness centrality was increased in the anterior brain areas, and decreased in the posterior regions, which resulted in an “anterior shift” of the hubs. Such an anterior shift of hub nodes has also been reported in frontotemporal dementia (FTD) (Franciotti et al., 2022). A loss of functional node degree, local and global efficiency was also reported in an EEG study using Granger causality as a measure of directed coupling (Franciotti et al., 2019). A loss of network integration was also found by Das and Puthankattil (2020). However, during a working memory task MCI subjects may actually show an increase in network integration and centrality (Fodor et al., 2021).

### MEG

An early MEG study showed a loss of functional connectivity in the alpha and beta band in AD (Stam et al., 2009). Of interest, the network changes in the AD group could be replicated by simulating a targeted attack on high degree nodes of the healthy network, and not by a random attack. This suggests that highly connected functional hubs may be more vulnerable in AD. A loss of functional connectivity in left temporal hubs was shown in a study using the synchronization likelihood as a functional connectivity measure (de Haan et al., 2012). More recent studies investigated MEG functional connectivity in source space. Yu et al. (2017) showed selective vulnerability of hubs (quantified with the hub disruption index) in the posterior default mode network in a multiplex network analysis. Hub vulnerability correlated with cognitive dysfunction and amyloid levels in the cerebrospinal fluid. A multilayer multifrequency approach to MEG network analysis was also used by Guillon et al. (2017). Hub failure was quantified by the decrease of a multiparticipation coefficient, especially in the cingulum and association cortex. In a study of directed functional connectivity, a lower outflow of information from the precuneus and visual cortex to the frontal and subcortical areas could be demonstrated in AD (Engels et al., 2017).

### Hub Involvement and Cognitive Dysfunction in AD

In view of the central position of hub nodes in communication networks in the brain, one would expect that damage to hubs would be correlated with cognitive dysfunction in AD. There is considerable evidence that this is the case in studies across different modalities. Cognitive dysfunction has been associated with damage to temporal hubs in MEG (de Haan et al., 2012), loss of functional connectivity of hubs and long-distance connections in resting-state fMRI (Liu et al., 2014b; Sui et al., 2015; Wang et al., 2015; Wang et al., 2021b; Zhang et al., 2021) and loss of structural connectivity in the rich club (Drenthen et al., 2022). Loss of network organization and cognitive dysfunction are also related to the APOE e4 allele (Wang et al., 2015).

### Conclusion

There is extensive evidence for early and relatively selective damage to hub nodes, in particular, the posterior cingulum and the precuneus, in AD. This selective hub damage is closely related to the local accumulation of amyloid-beta and tau. Targeted attack to highly connected and highly active hub nodes has also been demonstrated in simulation studies (Aerts et al., 2016; de Haan et al., 2012; Li et al., 2016; Stam et al., 2009). This link between high connectivity, high activity, and vulnerability to damage has been coined “activity dependent degeneration” (de Haan et al., 2012). Furthermore, when the overloaded hubs start to fail, they will lose their connections, and network traffic will be redirected to the next hubs in line (Hillary & Grafman, 2017; Watanabe et al., 2021). This could give rise to a scenario of cascading hub overload and failure (Jones et al., 2016; Stam, 2014). In fact, as will be discussed in the next section, hub overload and failure could be a very general scenario for brain network failure that is not limited to AD.

## HUB OVERLOAD AND FAILURE: A FINAL COMMON PATHWAY FOR NETWORK FAILURE IN NEUROLOGICAL DISEASE?

As we have seen there is extensive evidence for involvement of hubs in a variety of neurological disorders despite the fact that they are assumed to have a very different nature of the underlying pathophysiological process and distribution of the pathological lesion or lesions. An important question is if these apparently rather different conditions can be understood within a single comprehensive framework based upon the central role of hub nodes. Here I will examine to what extent the previously introduced scenario of “hub overload and failure” can provide such a framework (Stam, 2014).

I assume that a scenario of brain network failure starts with a lesion, or a number of lesions distributed throughout the brain. This lesion could be a stroke, an epileptic focus, or one or more MS plaques in nonhub areas of the brain. The assumption is that, whatever the detailed pathology of the initial lesion or lesions, a redistribution of network traffic and load to other brain areas will occur. This redistribution is expected to be proportional to the connectivity of the affected nodes, so the most highly connected hubs such as the precuneus and posterior cingulum will take the largest share of the increased load. Since we are dealing here with nodes that are natural hubs in healthy brain networks, I will refer to them as preexisting or physiological hubs. This scenario of network traffic redistribution will lead to a phase of hub overload: increased activity and metabolism, as well as increased functional connectivity. If the initial lesion or lesions recover, this hub overload and hyperconnectivity can be transitory, and the normal situation can be restored. However, if the hub overload is excessive, or lasts too long, structural damage to the hub and its connections is likely to occur. This hub failure will be associated with loss of activity, diminished structural and functional connectivity, atrophy and a reduced capacity of the hub to handle network traffic. It is to be expected that hub failure will have a large detrimental effect on network organization and information processing, resulting in general cognitive dysfunction.

**Figure 2.**
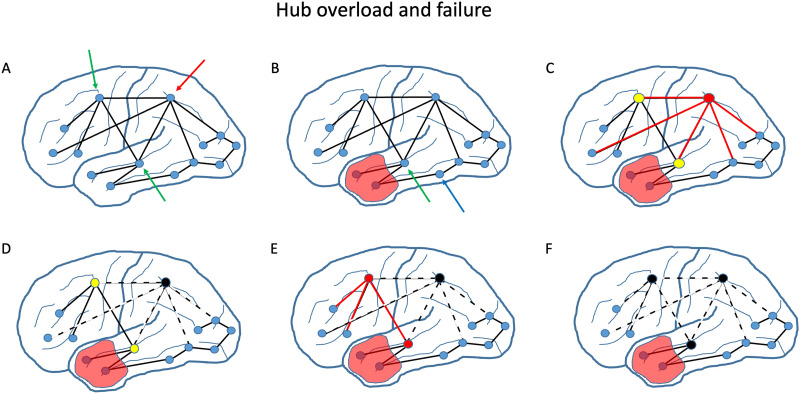
Hub overload and failure. Schematic illustration of proposed hub overload and failure scenario: (A) Under normal conditions the brain network has many nodes with relatively low degree and a few nodes with higher degrees such as the nodes indicated by the green arrows (degree 4) and the red arrow (degree 5). Traffic on the network (action potentials propagated along axons connecting spatially separate brain regions) is distributed proportionally to degree, so the more highly connected hub nodes handle more of the traffic than the lower degree nodes. (B) When part of the brain is damaged, such as the area indicated in red, the traffic is redistributed over the remaining healthy nodes. For instance, the length two path between the nodes indicated by the blue and the green arrow no longer exists, and is replaced by a length three path, which in this case involves the highly connected degree 5 hub node. (C) Traffic redistribution is proportional to node degree. The highest degree node with degree 5 shows the greatest increase in activity (firing rates of neurons in this brain region) and functional connectivity to its neighbors, indicated by the red colors. The slightly lower degree 4 nodes show a smaller increase in activity. (D) When the extra load on the hub is too high and/or persists too long, this hub will be damaged. As a consequence, its activity and functional connectivity will decrease, and network traffic is now redirected to the next hubs in line indicated by yellow. (E) Now, these new, pathological hubs will show, in turn, an increased level of activity and hyperconnectivity to their neighbors. (F) If these new hubs are overloaded and fail, they will also end up with low levels of activity and connectivity. In this way, damage can cascade through the whole network, severely damaging its organization and function.

Importantly, if the highest degree nodes start to fail, there will be a redistribution of network traffic to the next hubs in line. These nodes will subsequently increase their level of activity and functional connectivity. These nodes are referred to as pathological hubs, since they only emerge under abnormal conditions. Again, this could be a transitory phase with only functional changes, but it could also result in structural damage if the load increase is too severe and lasts too long. This is a scenario of *cascading failure*, where hubs become overloaded, get damaged, and pass the load to other nodes, which then may become overloaded and so on. Loss of the highest degree nodes, and redistribution of network traffic to other nodes, will results in a “hub shift” or reorganization of the brain network. A schematic overview of the hub overload and failure scenario is shown in Figure 2.

A number of comments can be made about this putative scenario. An important characteristic is that the specific nature of the pathology in the early phase is largely irrelevant for the cascading hub failure in the later phases. I have discussed four different neurological disorders as examples, but hub overload and failure have also been reported in several other conditions such as coma, Parkinson’s disease, traumatic brain injury, and glioma (Achard et al., 2012; Bagarinao et al., 2022; Fagerholm et al., 2015; Mandal et al., 2020). What matters is whether the initial pathology is severe enough to produce a significant redistribution of traffic on the network. The hub overload and failure scenario represents a kind of nonspecific final common pathway of many different types of neurological disorder. A second point is that this scenario provides one explanation for the selective vulnerability of hubs. If the redistribution of network traffic is proportional to node centrality, the nodes that already have the highest levels of activity and metabolism to begin with are expected to have to deal with the largest additional load in the case of damage elsewhere in the brain. Most likely this will increase the probability of failure.

The prediction is that an early phase of increased hub activity and increased functional connectivity will precede a possible later phase with structural hub damage. This early phase of increased activity and hyperconnectivity has been described in a wide variety of disorders (Delli Pizzi et al., 2019; Hillary & Grafman, 2017; Roger et al., 2020). However, there is disagreement about its interpretation. Sometimes it is considered to be a positive phenomenon that reflects active compensation of function loss elsewhere in the brain (Eijlers et al., 2017; Meijer et al., 2017). On the other hand, it can also be viewed as a manifestation of disinhibition and a disrupted excitation/inhibition balance (de Haan et al., 2012). There is increasing evidence that network hyperexcitability, which has been associated with toxic effects of amyloid-beta, is an important feature of the pathophysiology of Alzheimer’s disease and other types of neurodegeneration (Altuna et al., 2022). This is an important development from a clinical point of view since hyperexcitability can be detected with EEG and MEG, and could respond to treatment with anti-epileptic drugs (Csernus et al., 2022). Within the framework of the hub overload and failure scenario, it makes sense to interpret abnormal activation and hyperconnectivity of hubs as an early pathological phenomenon that should be treated to prevent subsequent structural damage to hub nodes. Furthermore, this scenario predicts that measures of hub functional hyperconnectivity could be potential biomarkers of hyperexcitability (Cuesta et al., 2022; Ranasinghe et al., 2022; Stam et al., 2023).

The proposed scenario predicts that failure of the physiological hubs can result in the emergence of pathological hubs elsewhere in the brain. There is considerable evidence that this kind of network reorganization occurs in a variety of brain disorders (Engels et al., 2015; Jin et al., 2015a; Llufriu et al., 2016; Nissen et al., 2016; Pagani et al., 2020; van Diessen et al., 2013b). An important point is that network reorganization is probably a relatively late phase where structural damage has already occurred to part of the network and where one can expect cognitive dysfunction. The emergence of pathological hubs could have a special significance in the context of severe focal epilepsy. Here, newly emerged pathological hubs, especially if they arise in the vicinity of the epileptic focus, could play a role in the spreading of seizure activity to the rest of the brain. This scenario is clinically important since it suggests that surgical removal or disconnection of the pathological hubs, even if they are located at some distance of the focus, could improve the surgical outcome after epilepsy surgery (Ramaraju et al., 2020; Zubler et al., 2015).

## CONCLUSION AND FUTURE PROSPECTS

The scientific understanding of complex networks has increased enormously in the past few decades and has reached a level where it can be applied fruitfully to the study of brain networks in health and disease. The clinical relevance of understanding brain networks is particularly clear in the case of hubs, highly connected and active brain areas that are sensitive to damage in a wide variety of neurological disorders. This has been illustrated by the examples of epilepsy, stroke, multiple sclerosis, and Alzheimer’s disease. Each of these examples illustrates the potential clinical relevance of hub involvement. In epilepsy, identification of pathological hubs could improve the optimal choice of resection areas in epilepsy surgery. In stroke, involvement of hubs in the lesion may predict the likelihood of recovery from poststroke cognitive dysfunction. In multiple sclerosis, network reorganization is also predictive of present and future cognitive problems. Finally, in Alzheimer’s disease, hub damage is closely related to the pathophysiological process, in particular, the deposition of amyloid-beta and tau and the occurrence of network hyperexcitability. This may open up opportunities for the development of new biomarkers for the detection and treatment of hyperexcitability in Alzheimer’s disease. Many empirically observed features of selective damage to hub nodes in a variety of neurological disorders can be understood within the framework of hub overload and failure. In future studies this framework could be used to develop new, testable hypotheses about brain network involvement in neurological disease and may suggest new approaches to their treatment.

## AUTHOR CONTRIBUTIONS

Cornelis Jan Stam: Conceptualization; Data curation; Writing – original draft; Writing – review & editing.

## TECHNICAL TERMS

Edge: Link or connection between two network nodes.
Graph: Abstract mathematical representation of a network as a set of nodes connected by a set of links.
Small-world network: Graph characterized by combination of short average path length and high clustering coefficient.
Connectivity: Physical connections between two brain regions (structural connectivity) or statistical relation between time series of activity recorded from two brain regions (functional connectivity).
Scale-free network: Graph with a power law degree distribution.
Hub: Network node with a high score on one or more measures of centrality.
Centrality: Measure of the relative importance of a node in a network.
Degree: Number of links connected to a node.
Path length: Number of edges that have to be travelled to get from one node to another in a graph.
Eigenvector centrality: Measure of the importance of a node that takes into account the importance of its neighbors, and the importance of their neighbors and so on.
Connector hub: Hub with many connections to other modules in the network.
Provincial hub: Hub with connections within its own local module.
Rich club: Set of hubs which are more strongly connected to each other than could be expected on the basis of their degree.
Efficiency: Inverse of path length. Measure of distance between nodes.
Default mode network: Resting-state network consisting of interconnected hubs in frontal, parietal and temporal brain areas.
Resting-state network: Set of brain regions more strongly connected to each other than to the rest of the brain. Usually in the context of resting-state fMRI.
